# Generating distant analogies facilitates relational integration: Intermediary role of relational mindset and cognitive load

**DOI:** 10.3389/fpsyg.2022.1012081

**Published:** 2022-09-13

**Authors:** Xuesong Du, Pei Sun

**Affiliations:** Department of Psychology, School of Social Science, Tsinghua University, Beijing, China

**Keywords:** distant analogy generation, relational integration, relational reasoning, relational mindset, cognitive load

## Abstract

Relational integration is essential for learning, working, and living, as we must encode enormous volumes of information and extract their relations to construct knowledge about the environment. Recent research hints that generating distant analogies can temporarily facilitate learners’ state-based relational integration. This study aimed to investigate the internal mechanism underlying the facilitation effect and preliminarily confirm its application in education. First, we adopted the classical n-term premise integration task (Experiment 1a) and the Latin Square Task (Experiment 1b) to explore the robustness of the facilitation effect. Then we employed an emerging multidimensional relational reasoning task to further explore the internal mechanism underlying this facilitation effect (Experiment 2). Finally, we verified the practical role of the facilitation effect in learning the interaction concept in statistics (Experiment 3). The results showed that generating distant analogies did facilitate students’ relational integration performance, both in classical cognitive tasks and in a practical learning task, and a relational mindset and cognitive load play an intermediary role in the facilitation, supporting the cognitive load theory. The results suggest that generating distant analogies can be a useful warm-up activity to assist educators in promoting students’ relational integration.

## Introduction

According to James (1890), we would be imprisoned in a world of isolated stimuli if not for the ability to perceive relevant relations among the objects of our perception, even when they are separated by time and space (p. 502 in Volume I and p. 347 in Volume II). Relational reasoning is an essential predictor of individual academic achievement and other important life variables (Goldwater and Schalk, 2016; Dumas and Dong, 2019), contributing significantly to learning (Knowlton et al., 2012), creativity (Weinberger et al., 2016; Green, 2017), and social development (Green et al., 2017). Understanding how to better perform relational reasoning is especially important in today’s information-rich age (Braasch et al., 2013). However, research about whether and how relational reasoning could be facilitated is limited. Recent research shows that generating distant analogies can facilitate learners’ relational reasoning (Vendetti et al., 2014; Andrews and Bohadana, 2018; Andrews and Vann, 2019; Goldwater and Jamrozik, 2019); however, the internal mechanism of this facilitation effect is unclear.

### Ability-based and state-based facilitation in relational reasoning

Relational reasoning, one of the most fundamental human cognitive abilities, can be defined as an individual’s ability to notice critical similarities and differences between seemingly unrelated information (Alexander et al., 2016a,b). Research in cognitive science (Sternberg, 1977; Gick and Holyoak, 1980) and cognitive neuroscience (Green et al., 2010, 2012; Whitaker et al., 2017) consistently shows that there are three crucial processes in relational reasoning, i.e., controlled semantic retrieval, inhibitory control, and relational integration, with relational integration being the core cognitive component (Whitaker et al., 2017).

Relational integration is the ability to mentally link variables related to goal-directed behavior, and it underpins various higher-order cognitive abilities, including reasoning, categorization, planning, and problem-solving (Bunch et al., 2007; Andrews et al., 2013; Andrews and Mihelic, 2014). Its two basic qualities are domain-generality and effortfulness. Relational integration involves a variety of tasks, such as sentence comprehension and transitive reasoning, and requires effort, which stems from relational complexity (Andrews et al., 2006).

Regarding how to facilitate relational reasoning, most previous studies focused on ability-based facilitation, which aims to improve learners’ ability to reason with relations (Simms et al., 2018). There are at least three types of ability-based facilitation: formal schooling, curricula to enhance reasoning, and laboratory-developed reasoning training programs. In contrast, state-based facilitation, i.e., the tendency to notice relationships when solving problems, has been studied to a limited extent. Bliznashki and Kokinov (2010) discovered that completing a task that required participants to analyze relations unconsciously would enable them to complete subsequent tasks in a more relational manner, demonstrating that participants’ cognitive states might be temporarily altered. Here, we argue that short-term facilitation of relational reasoning in the state is more flexible, economical, and easier to implement than ability-based facilitation.

### Generating distant analogies as a potential means of facilitation

Generating distant analogies is a promising method of facilitating learners’ relational reasoning from the perspective of cognitive state. Analogy is the cognitive process of mapping relations from one known context to another unknown context to infer new conclusions or explanations (Gentner, 2016). Semantic distance is the distance in semantic space between the corresponding representations of the source analog and the target analog. Near analogies can be made by matching consistent relations, for example, furnace:coal: woodstove:wood (a furnace burns coal, just like a woodstove burns wood), whereas distant analogies necessitate making evaluations across domains or generating a more abstract relation, for example, furnace:coal: stomach:food [a furnace burns coal, just like the stomach “burns” (digests) food].

Recent studies have shown that generating distant analogies could induce temporary promotion in relational integration. Vendetti et al. (2014) provided the first empirical demonstration that when participants solved the distant analogies first, they were more inclined to respond based on the same role that objects played in different scenes rather than on common visual features shared by the objects in the scene mapping task, indicating that generating distant analogies, rather than simply evaluating them, could facilitate relational reasoning. Generating distant analogies could facilitate participants’ relational integration in the more complex quaternary relations in the n-term premise task (a classic relational integration task, similar to linear syllogisms; Andrews and Bohadana, 2018). Andrews and Vann (2019) showed that generating distant analogies could influence participants’ belief-based reliance on relational processing in valid unbelievable problems. Research by Goldwater and Jamrozik (2019) demonstrated that solving distant analogies would increase people’s analogy retrieval before information was coded in a problem-solving task.

It is still unknown whether other tasks similar to the distant analogy generation task could promote relational integration as well, such as the distant analogy evaluation task. There have been few studies on the distant analogy evaluation task. Neuroimaging studies have provided some findings. Using fMRI, Green et al. (2010, 2012) demonstrated that distant analogies were closely related to relational integration. They found that as semantic distance increased, both evaluating the validity of analogies and generating the missing items of the analogies resulted in increased activity in the left prefrontal cortex (rostrolateral prefrontal cortex, a brain region involved in relational integration in numerous reasoning tasks), implying the possibility of facilitating relational integration in both the evaluation task and the generation task. Given the findings in neuroimaging research, we sought to explore whether the distant analogy evaluation task could also facilitate relational integration in the same way as the distant analogy generation task.

### Internal mechanism of the facilitation effect

A relational mindset is a general tendency to seek out and prioritize relational information (Holyoak and Thagard, 1996). The overall relational mindset emerges when individuals actively identify and construct similar relations, leading to more relational responses in subsequent tasks (Brown and Kane, 1988; Bliznashki and Kokinov, 2010; Goldwater and Markman, 2011). The demands of the previous task can alter people’s sensitivity to relational information and affect subsequent task performance. Several recent studies on adults (Vendetti et al., 2014) and children (Simms and Richland, 2019; Murphy et al., 2021) have found that generating distant analogies, as a task for generating relational information, may trigger a relational mindset. For example, Vendetti et al. (2014) found that completing the distant analogy generation task could induce a “general relational mindset,” which encouraged learners to pay more attention to relations and in turn helped individuals to respond more based on relations in a subsequent task with unrelated material. Therefore, the relational mindset may play a mediating role between generating analogies and relational integration.

Learning tasks should be designed to minimize cognitive processing unrelated to learning and release working memory capacity to maximize the working memory resources available for learning-related tasks. Cognitive load is the working memory load experienced when completing a specific task (Kalyuga, 2011; Sweller et al., 2011). Learning may be inhibited if the cognitive load exceeds the available working memory capacity. There are two types of cognitive load, intrinsic and extraneous cognitive load (Sweller, 1994). Intrinsic cognitive load is the relevant, necessary load required to achieve a specific learning goal, determined by the nature of the learning material and the learner’s knowledge. Extraneous cognitive load is not related to the achievement of learning goals; it is caused by the cognitive activities that learners need to perform due to the specific design of the learning task. Cognitive load theory later introduced germane cognitive load to explain the positive effects of additional learning activities (Sweller, 2010; Sweller et al., 2019). While these activities increase the demands on working memory, they promote learning, for example, by prompting learners to self-interpret or increase task variability when learning from examples (Sweller et al., 1998).

Since relational processing is effortful, which will impose a high load on individual’s limited cognitive resources (Halford et al., 1998), it is necessary to consider how to reduce learners’ cognitive load when designing interventions (Gray and Holyoak, 2020, 2021). According to the “*environmental-organizing-and-linking principle*” of cognitive load theory (Sweller et al., 1998; Sweller and Sweller, 2006), the external environment provides cues that trigger relevant schemas in long-term memory, prompting individuals to use schemas to produce actions suitable for the environment. When schemas are introduced from long-term memory into working memory, they only need to be processed as one element, which can reduce the occupation of working memory. We assume that after completing a task of generating relations, it may cause individuals to continue generating relations in subsequent tasks to adapt to the environment, correspondingly reducing learners’ cognitive load. Therefore, cognitive load may serve as another possible internal factor.

### Possibility of applying the facilitation effect in learning

Introducing relational thinking in the classroom may aid students in developing their conceptual understanding and problem-solving skills because many concepts taught in theoretical and practical disciplines are relational in nature (English and Halford, 2012; Singley and Bunge, 2014; Vendetti et al., 2015). For instance, many college students struggle to comprehend and interpret statistical interactions. This difficulty may stem from the complex relations involved in interactions. Students may find it easier to understand concepts with high relational complexity if they first come up with distant analogies. To our knowledge, no controlled laboratory research has demonstrated the influence of generating distant analogies on immediate relational reasoning outcomes using authentic learning materials, excluding confounding variables such as prior knowledge. We speculated that having learners complete a distant analogy generation task prior to an instructional task related to relational reasoning could improve their learning outcomes.

### Current study

In this study, we explored the robustness of the distant analogy generation task in facilitating relational integration and the possibility that the distant analogy evaluation task could promote relational integration. We employed two classic relational integration paradigms, the n-term premise integration task (Experiment 1a) and the Latin Square Task (LST) (Experiment 1b). When solving distant analogy generation problems, participants need to search for potential options based on semantic relations and determine the answer by evaluating the relations between A and B and the common constraints of item C. In contrast, evaluating complete analogies bypasses generating answers to complete the analogy structure. Therefore, we predicted that only the distant analogy generation task would promote relational integration, while the distant analogy evaluation task would not. We adopted the multidimensional relational reasoning task (MRRT) in Experiment 2 to further explore the internal mechanism underlying the facilitation effect, investigating the role of the relational mindset and cognitive load. We hypothesized that the distant analogy generation task could ultimately facilitate relational reasoning by triggering individuals’ relational mindset, which would then reduce their cognitive load. Experiment 3 aimed to demonstrate how generating distant analogies could influence the learning processes and outcomes in an experimental setting using a real learning task. It was based on a randomized design consisting of two groups: a distant analogy generation condition and a distant analogy evaluation condition. We assumed that the distant generation group would have better performance than the distant evaluation group.

## Experiment 1a

The purpose of Experiment 1a was to explore the robustness of generating distant analogies and the possibility of evaluating distant analogies by investigating the influence of **priming task type (generation and evaluation)** and **semantic distance (control, near, and distant)** on relational integration. We adopted the n-term premise integration paradigm used in previous studies (Andrews et al., 2006; Andrews and Bohadana, 2018) and set two relational complexity levels: the ternary level and the quaternary level. We hypothesized that only the distant analogy generating task, and not the distant analogy evaluation task, would foster relational integration. Specifically, if the priming task was the generation task, the distant group would perform significantly better than the near group and the control group, while if the priming task was the evaluation task, there would be no significant difference between the distant group and the other two groups.

### Materials and methods

#### Participants

Two hundred and twenty college students were recruited, with 109 males and 111 females. Their mean age was 20.95 years (SD = 2.31, range: 18–28). The sample size was chosen based on an *a priori* power calculation for an effect size of η^2^ = 0.07 (Vendetti et al., 2014; Andrews and Bohadana, 2018; Andrews and Vann, 2019) for power = 99% and Type I error rate = 5%. We randomly allocated participants to one of six groups: **GC** (Generation/Control; *n* = 36), **GN** (Generation/Near; *n* = 37), **GD** (Generation/Distant; *n* = 38), **EC** (Evaluation/Control; *n* = 36), **EN** (Evaluation/Near; *n* = 37), or **ED** (Evaluation/Distant; *n* = 36). All of the experiments received approval from the relevant ethics committee. Participants provided their written informed consent to participate in this study.

#### Materials

##### Priming task

Considering that the participants were all native Chinese-speaking college students, all experimental materials were translated into Chinese by two graduate students majoring in psychology and one graduate student majoring in English. The materials in the priming task were mainly drawn from Green et al. (2010). Given possible cultural differences, 30 college students who did not participate in the main experiment were asked to rate word familiarity (1 = *very unfamiliar*, 7 = *very familiar*), the rationality of word pair relations (1 = *very unreasonable*, 7 = *very reasonable*), and the semantic distance between word pairs (1 = *very near*, 7 = *very distant*) on a seven-point Likert scale. Items with familiarity, rationality, and semantic distance ratings of more than five were chosen as formal materials. Eighty analogy generation problems were identified for the formal experiment, with 40 near problems and 40 distant problems. Each problem was presented in the form of A:B: C:__ (“__” represented the missing D term). The first half of the near and distant problems were identical, with the difference being in the second half—for example, in the near problem furnace:coal: woodstove:__ and the distant problem furnace:coal: stomach:__. The **GN condition** completed the near analogy problems, while the **GD condition** completed the distant analogy problems. Except for the difference in core components (without analogy), the cognitive processes involved between the control group and the experimental group were as consistent as possible. Referring to previous literature (Andrews and Bohadana, 2018; Goldwater and Jamrozik, 2019), we chose the word generation task as GC and the semantic distance evaluation task as EC. The items for the **GC condition** were drawn from problems in the GN condition (A:B: C1: __) and the GD condition (A:B: C2:__). We extracted A:B, C1:D1, and C2:D2 as a version of the material, with each problem adding a word that had semantic relation to the other two words, e.g., “furnace coal ironmaking ?”, “woodstove wood heat ?”, “stomach food digest ?” This resulted in three versions of word generation problems. Participants in the control group were randomly assigned to one version.

The items for the evaluation task were basically the same as those for the generation task, with the exception that the format was A:B: C:D. The first half of the problems (A:B) was the same for the GN and GD conditions, with the difference being in the second half (C:D). The near analogy problems were like furnace:coal: woodstove:wood, while the distant analogy problems were like furnace:coal: stomach:food. Participants needed to judge whether the relations between A:B and C:D were consistent. There were 40 problems in the **EN and ED condition** (30 consistent problems and 10 inconsistent problems). The items for the **EC condition** were essentially the same as those for the GC condition, but the task requirement was different. Participants were asked to assess the semantic distance between the given three words, e.g., “furnace, coal, ironmaking,” on a seven-point scale (1 = *very near*; 7 = *very distant*) according to their understanding. There were three versions of the semantic distance assessing materials, and participants in the control group were randomly assigned to one of them.

##### N-term premise integration task

The n-term premise integration task (Andrews et al., 2006; Hansell et al., 2015) is a linear syllogistic reasoning task. Participants were presented with a set of premises (e.g., B < G, G > K, B < K), and they needed to integrate these premises to construct descending sequences of letters (e.g., G > K > B). There were two levels of relational complexity: ternary and quaternary. Ternary problems required integration of three premises (containing three letters); quaternary problems had higher relational complexity and required integration of four premises (containing four letters). We also applied the following controls to reduce the interference of extra variables on the experimental results: Letters that resembled Arabic numerals (I, O, S, and Z) and the letter W were removed (participants could use the verbal repetition strategy, and W was pronounced with three syllables); the frequency of each letter in the problems was approximately the same; at most, only one letter was repeated in two adjacent problems. For the ternary and quaternary conditions, there were two practice problems and 10 formal problems, respectively. The maximum score in each condition possible was 10.

##### Fluid intelligence

Fluid intelligence was measured using a short (12-item) version of Raven’s Progressive Matrices (RPM) (Arthur et al., 1999), a commonly used test of fluid intelligence. The test presented a series of 3 × 3 matrices, with each cell having a shape and only the bottom right cell being blank. Participants were asked to observe the pattern of variation in the rows and/or columns of each matrix and to select the shape from eight options to correctly complete the matrix based on the relational rules they found. The task was limited to 11 min, and no feedback was given. The maximum score possible was 12.

#### Procedure

Participants completed all tasks individually on a computer, using the keyboard to input their responses. During the first phase, participants were asked to perform a priming task. The GC group completed the word generation task, the GN group completed the near analogy generation task, and the GD group completing the distant analogy generation task. The EC group completed the semantic distance assessing task, the EN group completed the near analogy evaluation task, and the ED group completing the distant analogy evaluation task. All of the participants completed two practice problems to familiarize themselves with the task rules and then completed the 40 problems in the formal part. The task had no time limit, and there was no feedback. Participants were then asked to rate the difficulty of the priming task on a seven-point scale (1 = *very easy*; 7 = *very difficult*). The second phase was the n-term premise integration task. To ensure the difficulty of the task and to prevent participants from writing down their answers to the problems directly, the problem and answer pages were set as separate pages. Participants were required to arrange the letters in descending order (no drafts were allowed) and then enter their answers on the next page. The task had no time constraints. During the third phase, each participant had to complete the fluid intelligence test with a time limit of 11 min. The overall duration of Experiment 1a was approximately 35 min.

#### Design

Experiment 1a implemented a 2 (relational complexity: ternary, quaternary) × 2 (priming task type: generation, evaluation) × 3 (semantic distance: control group, near, distant) mixed design, with relational complexity as a within-subject factor and priming task type and semantic distance as between-subject factors. The dependent variables were task scores and solution time. The covariates were the participants’ ratings of the difficulty of the priming task and their fluid intelligence scores.

### Results

#### Scores

**FIGURE 1:**
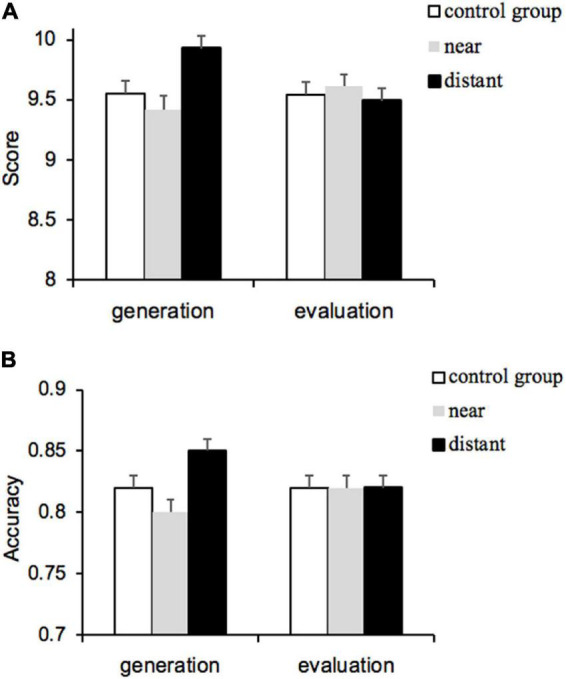
Score and accuracy in Experiment 1. **(A)** Mean score in Experiment 1a and **(B)** accuracy in Experiment 1b. Error bars indicate standard errors.

Given the possible differences in the cognitive effort spent by participants in completing the priming task and in the participants’ fluid intelligence, we took these two variables as covariates and the task scores as the outcome variable. A 2 (relational complexity: ternary, quaternary) × 2 (priming task type: generation, evaluation) × 3 (semantic distance: control group, near, distant) repeated measures ANOVA was conducted. The main effect of relational complexity was significant (*F*_(1_, _212)_ = 18.63, *p* < 0.001, partial η^2^ = 0.08), and the scores for the ternary condition (*M* = 9.66, SE = 0.04) were significantly higher than those for the quaternary condition (*M* = 9.52, SE = 0.06), indicating that the manipulation of relational complexity was effective. There were no significant interactions between relational complexity and other factors. The main effects of priming task type (*F*_(1_, _212)_ = 1.03, *p* = 0.312, partial η^2^ = 0.01) and semantic distance (*F*_(2_, _212)_ = 1.92, *p* = 0.150, partial η^2^ = 0.02) were not significant; however, a significant interaction between these two factors was found (*F*_(2_, _212)_ = 4.82, *p* = 0.009, partial η^2^ = 0.04), as shown in Figure 1A. The simple effect analysis revealed no significant differences in scores among the control group (*M* = 9.54, SE = 0.11), near group (*M* = 9.61, SE = 0.10), and distant group (*M* = 9.49, SE = 0.11) for the evaluation task. However, for the generation task, scores in the distant group (*M* = 9.93, SE = 0.10) were significantly higher than those in the near group (*M* = 9.42, SE = 0.11), *t*_(73)_ = 3.43, *p* = 0.011, Cohen’s *d* = 0.23. Meanwhile, scores in the distant and control group (*M* = 9.55, SE = 0.11) were not significantly different, *t*_(72)_ = 2.57, *p* = 0.162, Cohen’s *d* = 0.17. These results indicated that the evaluation task had no facilitation effect on relational integration at all levels of relational complexity, whereas the facilitation effect of the generation task on relational integration increased with semantic distance, i.e., only generating distant analogies could promote relational integration, while evaluating distant analogies did not work.

#### Solution times

Solution times were based on correct responses only (Andrews and Bohadana, 2018), which were tested using the same analysis as task scores. The 2 × 2 × 3 ANOVA results showed that the main effect of relational complexity was significant (*F*_(1_, _212)_ = 57.48, *p* < 0.001, partial η^2^ = 0.21), as the solution times for the ternary condition (*M* = 132.61, SE = 2.11) were significantly shorter than those for the quaternary condition (*M* = 216.81, SE = 4.57). There were no significant interactions between relational complexity and other factors. The main effects of priming task type (*F*_(1_, _212)_ = 0.96, *p* = 0.330, partial η^2^ = 0.004) and semantic distance (*F*_(2_, _212)_ = 1.76, *p* = 0.175, partial η^2^ = 0.02) were not significant, and there was no significant interaction between priming task type and semantic distance (*F*_(2_, _212)_ = 0.44, *p* = 0.642, partial η^2^ = 0.004).

### Discussion

Experiment 1a explored whether or not generating and evaluating distant analogies facilitated relational integration. We found that generating distant analogies was effective in boosting participants’ scores on the n-term premise integration task compared to generating near analogies, confirming the facilitation effect of generating distant analogies, as we expected. In contrast, such a facilitation effect was not found when evaluating distant analogies. Experiment 1a used the same paradigm as Andrews and Bohadana (2018) to validate the facilitation effect of generating distant analogies and to preliminarily rule out the possibility of the evaluation task as the priming task, expanding the conclusions of Andrews and Bohadana (2018). To consolidate our findings, we used another relational integration paradigm in Experiment 1b.

## Experiment 1b

Experiment 1b aimed to provide integrative evidence by corroborating the findings of Experiment 1a with a recently developed relational integration paradigm (the LST, Birney et al., 2006; Hearne et al., 2020). We set two task variables: **relational complexity** and **processing step**. The higher the level of relational complexity, the more processing steps, and the more cognitive effort the relational integration task consumed. Based on the findings of Experiment 1a, we expected that only generating distant analogies would facilitate relational integration, but not evaluating distant analogies, i.e., there would be an interaction between priming task type and semantic distance.

### Materials and methods

#### Participants

Two hundred and fifteen college students were recruited, with 111 males and 104 females. Their mean age was 20.93 years (SD = 2.33, range: 18–28). The sample size was estimated based on an *a priori* power calculation for an effect size of η^2^ = 0.07 (Vendetti et al., 2014; Andrews and Bohadana, 2018; Andrews and Vann, 2019) for power = 99% and Type I error rate = 5%. We randomly allocated participants to one of six groups: GC (*n* = 35), GN (*n* = 36), GD (*n* = 34), EC (*n* = 36), EN (*n* = 37), or ED (*n* = 37).

#### Materials

##### Priming task and fluid intelligence

The materials for the priming tasks and fluid intelligence test were the same as those in Experiment 1a.

##### Latin square task

The LST is a non-verbal task (Hearne et al., 2020) that contains four geometric shapes (triangle, square, circle, and cross). Each item contains a 4 × 4 matrix containing geometric shapes, blank cells, and a “?” (indicating the target location). The reasoner needs to infer what shape should be at the “?” according to the task rules (each geometric shape can only appear once in each row and each column). There are two factors that affect the difficulty of the LST. One is the relational complexity (binary, ternary, and quaternary). Binary problems require the integration of information across a single row or column, ternary problems require the integration of information across a single row and column, and quaternary problems are the most complex, requiring the integration of information across multiple rows and columns. The other factor is the processing step (one-step and two-step). The “one-step” problems lead directly to the answer, whereas the “two-step” problems require one relation to be solved first and then a second relation to be solved before deriving the answer. Therefore, to solve the second problem, the reasoner must remember the answer to the initial relation, thus increasing the reasoner’s working memory load. We utilized the LST program in Hearne et al. (2020), written using Matlab2014b (The MathWorks, Inc., Natick, MA, United States) and the Psychtoolbox (Brainard and Vision, 1997). Stimuli were presented on a CRT screen (100 Hz refresh rate, 1024 × 768 pixels) with a viewing distance of 70 cm, and key responses were recorded.

#### Procedure

The procedure was the same as for Experiment 1a, except that the task in the second phase was replaced by the LST. Participants first practiced (12 trials) and then completed the formal task. The formal task contained 144 trials in total and consisted of two parts: 108 standard trials with six levels of 18 trials each and 36 control trials. In the control trials, the matrices had an asterisk (*) instead of a question mark (?). The number and spatial position of the geometric shapes, and all of the visual features in the control trials matched those of the standard trials. The only difference was that no reasoning was required. Each trial began with a fixation in the center of the screen (2,000 ms), followed by an LST problem (it remained on the screen for a maximum of 12 s and could be aborted by pressing the space bar if the participant knew the answer). This was followed by a fixation (1,000 ms) and a key response screen (participants needed to press the key within 5 s, with options from left to right on the screen corresponding to the “1,” “2,” “9,” and “0” keys; they pressed the key according to the inferred geometric shapes for the standard trials and pressed the space bar directly for the control trials). In the formal task, at the end of each block, the accuracy of the current block was fed back. The trials were given in pseudo-random order to ensure that trials with the same relational complexity did not occur consecutively. We were concerned with two indicators: accuracy and processing times (the time taken from the presentation of the problem to the time the participant pressed the space bar, i.e., the time spent on relation processing). It took roughly 30–40 min to finish the LST. Experiment 1b lasted around 60 min in total.

#### Design

Experiment 1b implemented a 3 (relational complexity: binary, ternary, and quaternary) × 2 (processing step: one-step and two-step) × 2 (priming task type: generation and evaluation) × 3 (semantic distance: control group, near, and distant) mixed design, with relational complexity and processing step as within-subject variables and priming task type and semantic distance as between-subject variables. The dependent variables were accuracy and average processing time. The covariates were the participants’ ratings of the difficulty of the priming task, their responses in control trials, and their fluid intelligence scores.

### Results

#### Accuracy

Considering that there may be differences between participants’ cognitive effort when they completed the priming task and in their fluid intelligence, we used these two variables and the accuracy in control trials as covariates, and the accuracy as the outcome variable. We conducted a 3 (relational complexity: binary, ternary, and quaternary) × 2 (processing step: one-step and two-step) × 2 (priming task type: generation and evaluation) × 3 (semantic distance: control group, near, and distant) repeated measures ANOVA. The main effect of relational complexity was significant (*F*_(2_, _318)_ = 39.33, *p* < 0.001, partial η^2^ = 0.16), with the accuracy for binary items (*M* = 0.96, SE = 0.003) being significantly higher than the accuracy for ternary items (*M* = 0.90, SE = 0.01) and quaternary items (*M* = 0.60, SE = 0.01), indicating that the higher the relational complexity, the more difficult the relational integration. The interaction between relational complexity and semantic distance was significant (*F*_(4_, _318)_ = 2.70, *p* = 0.044, partial η^2^ = 0.03). For binary and ternary items, there was no significant difference in the accuracy of different semantic distances (*ps* = 1.000). However, for higher relational complexity (quaternary items), the accuracy in the distant group (*M* = 0.63, SE = 0.01) differed significantly from that in the control group (*t*_(140)_ = 3.38, *p* = 0.028, Cohen’s *d* = 0.23) and the near group (*t*_(142)_ = 3.22, *p* = 0.049, Cohen’s *d* = 0.22), respectively. Meanwhile, there was no significant difference in the accuracy between the control group (*M* = 0.59, SE = 0.01) and the near group (*M* = 0.59, SE = 0.01). The main effects of processing step (*F*_(1_, _206)_ = 0.42, *p* = 0.516, partial η^2^ = 0.002) and priming task type (*F*_(1_, _206)_ = 0.18, *p* = 0.669, partial η^2^ = 0.001) were not significant. The main effect of semantic distance was significant (*F*_(2_, _206)_ = 3.57, *p* = 0.030, partial η^2^ = 0.03), with a significantly higher accuracy for the distant group (*M* = 0.84, SE = 0.01) than for the near group (*M* = 0.81, SE = 0.01) and a non-significant difference from the control group (*M* = 0.82, SE = 0.01). Importantly, a significant interaction between priming task type and semantic distance was revealed (*F*_(2_, _206)_ = 3.20, *p* = 0.043, partial η^2^ = 0.03), as shown in Figure 1B. For the evaluation task, there was no significant difference in the accuracy among the control group (*M* = 0.82, SE = 0.01), the near group (*M* = 0.82, SE = 0.01), and the distant group (*M* = 0.82, SE = 0.01). For the generation task, the accuracy in the distant group (*M* = 0.85, SE = 0.01) was significantly higher than that in the near group (*M* = 0.80, SE = 0.01), *t*_(68)_ = 3.50, *p* = 0.008, Cohen’s *d* = 0.24. Additionally, there was no significant difference between the accuracy in the control group (*M* = 0.82, SE = 0.01) and that in the distant group, *t*_(67)_ = 2.40, *p* = 0.259, Cohen’s *d* = 0.16. These results indicated that the evaluation task did not facilitate relational integration. In contrast, the generation of distant analogies could promote relational integration, and the facilitation effect of the generation task increased with semantic distance.

#### Processing times

Processing times were calculated across all trials, including correct trials and incorrect trials (Hearne et al., 2020). Trials with processing times shorter than 1 s and key response times longer than 5 s were excluded. A 3 × 2 × 2 × 3 repeated measures ANOVA similar to the accuracy data was conducted. The covariates were the priming task difficulty ratings, the processing times in the control trials, and the fluid intelligence scores, and the outcome variable was processing time. The main effect of relational complexity was significant (*F*_(2_, _406)_ = 31.64, *p* < 0.001, partial η^2^ = 0.13), and processing times for binary items (*M* = 4.39, SE = 0.07) were significantly shorter than those for ternary items (*M* = 7.19, SE = 0.10) and quaternary items (*M* = 9.75, SE = 0.08). There were no significant interactions between relational complexity and other factors. The main effect of processing step was significant (*F*_(1_, _206)_ = 26.58, *p* < 0.001, partial η^2^ = 0.11), with processing times of one-step items (*M* = 5.85, SE = 0.07) significantly shorter than those of two-step items (*M* = 8.37, SE = 0.08). The interaction between relational complexity and processing step was significant (*F*_(2_, _350)_ = 9.72, *p* < 0.001, partial η^2^ = 0.05). The difference in processing times between the two levels of processing step was greatest for the binary items, *t*_(214)_ = 39.92, *p* < 0.001, Cohen’s *d* = 2.72; followed by the quaternary items, *t*_(214)_ = 18.96, *p* < 0.001, Cohen’s *d* = 1.29; the ternary items had the smallest difference between the two processing step levels, *t*_(214)_ = 17.14, *p* < 0.001, Cohen’s *d* = 1.17. The main effects of priming task type (*F*_(1_, _206)_ = 0.11, *p* = 0.740, partial η^2^ = 0.001) and semantic distance (*F*_(2_, _206)_ = 0.08, *p* = 0.924, partial η^2^ = 0.001) were not significant. The interaction between priming task type and semantic distance was also not significant (*F*_(2_, _206)_ = 0.82, *p* = 0.444, partial η^2^ = 0.01). As in Experiment 1a, we did not observe an interaction between priming task type and semantic distance in processing times.

### Discussion

Experiment 1b examined again whether evaluating distant analogies could promote individuals’ relational integration performance in the LST. We discovered that evaluating distant analogies had no effect on individuals’ relational integration performance. For a comparison, in the distant condition, the generation task had a facilitative effect on relational integration. Consistent with the findings of Experiment 1a, Experiment 1b confirmed the facilitation effect of generating distant analogies while excluding the possibility that the distant analogy evaluation task facilitated relational integration. Experiment 1a and 1b both revealed that the presence of the “distant analogy” was insufficient to foster relational integration; “generation” serves as another indispensable component. In Experiment 2, we wished to delve further into the reason why the generation of distant analogies may facilitate relational integration compared to the evaluation task, that is, the internal mechanism of this facilitation effect.

## Experiment 2

Experiment 1 established the robustness of the facilitation effect for generating distant analogies but not for evaluating distant analogies: the previous findings were replicated in the classic paradigm (Experiment 1a, n-term premise integration task), and the same supporting evidence was obtained in the new paradigm (Experiment 1b, the LST). To further explore the internal mechanism underlying this facilitation effect, we employed an emerging MRRT in Experiment 2. This task was able to systematically examine the influence of different stimulus properties on relational reasoning, such as the number of premises and the number of dimensions, although this was not the primary purpose of the present study. Examining relational reasoning in the context of sentence judgments based on concrete language had higher ecological validity than Experiment 1a (letters) and Experiment 1b (geometric figures). We investigated two possible factors based on previous research and relevant theories: relational mindset and cognitive load. For comparison, we used the same two priming tasks: the **generating distant analogy task** and **evaluating distant analogy task**. We predicted that relational mindset and cognitive load would play a mediating role in the facilitation effect.

### Materials and methods

#### Participants

A total of 131 college students were recruited, with 59 male students and 72 female students. Their mean age was 20.87 years (SD = 2.40, range: 18–27). The current sample size was estimated based on an *a priori* power calculation for an effect size of Cohen’s *d* = 0.84 (Vendetti et al., 2014) for power = 96% and Type I error rate = 5%. We randomly allocated participants to the distant analogy generation group (GD; *n* = 67) and distant analogy evaluation group (ED; *n* = 64).

#### Materials

##### Priming task and fluid intelligence

The materials used in priming tasks (GD group and ED group) and the fluid intelligence test employed in Experiment 2 were the same as in Experiment 1a.

##### Relational mindset

We chose two tasks to measure the relational mindset, one was the scene mapping task and the other was the matching to sample task. The scene mapping task was often used to measure the degree of relational mindset (Vendetti et al., 2014; Goldwater and Jamrozik, 2019; Simms and Richland, 2019). To obtain more stable results, we also included the matching to sample task, which could also be used to measure the relational mindset (Gray and Holyoak, 2020). The first part was the scene mapping task (Markman and Gentner, 1993; Tohill and Holyoak, 2000; Kalkstein et al., 2020), with a total of 10 questions. Each question was presented with an upper and lower graph. In the upper graph, a certain object was circled, and participants were asked to select the “corresponding” or “matching” object among the options given in the lower graph. The lower graph included an option that was consistent with the role of the target object (based on relational similarity) and an option that was consistent with the shape of the target object (based on perceptual similarity). The second part was the matching to sample task (Goldstone et al., 1991), which consisted of eight questions. Each question was presented with the target picture on top and two options below it. One option had high perceptual similarity to the target figure but low relational similarity, whereas the other had low perceptual similarity to the target figure but high relational similarity. Participants had to choose the option that was “more similar” to the target figure. Selections based on relational similarity or perceptual similarity were available in both tasks. The maximum score was 18.

##### Cognitive load questionnaire

Cognitive load was measured using the cognitive load scale developed by Klepsch et al. (2017). The scale measures intrinsic cognitive load with two items (e.g., “For this task, many things needed to be kept in mind simultaneously”), extraneous cognitive load with three items (e.g., “During this task, it was exhausting to find the important information”), and germane cognitive load with two items (e.g., “I made an effort, not only to understand several details, but to understand the overall context”). All of the items were rated on a seven-point Likert scale (1 = *completely wrong*, 7 = *absolutely right*). The three components showed good consistency reliability (Cronbach’s alpha = 0.82 for intrinsic cognitive load; Cronbach’s alpha = 0.78 for extraneous cognitive load; Cronbach’s alpha = 0.75 for germane cognitive load). Sum scores of each of the three cognitive load measures were created.

##### Multidimensional relational reasoning task

Relation integration performance was measured by the MRRT (Cortes et al., 2021). Each problem consisted of two or three premises and a conclusion, and these problems systematically manipulated the following properties: number of premises (two or three), number of dimensions (one or two), and order of premises (continuous or discontinuous). Participants had to judge whether the conclusion was true according to the given premises. Participants were instructed to respond with “True” if the conclusion necessarily followed from the premises, “False” if the conclusion had to be wrong, or “Uncertain” if the given information was insufficient to determine the truth or falsehood of the conclusion. Given the length of the experiment and the difficulty of the task, 69 problems with “discontinuous” sequences were maintained, and nine easier problems were removed, leaving 60 problems for the formal experiment. The highest score was 60.

#### Procedure

Experiment 2 began with the priming phase, which used the same materials as Experiment 1a and 1b. The second phase was the relational mindset task. The instructions specifically emphasized that there was no standard answer. The task had no time constraints. The third phase was the MRRT. There were three practice problems and four formal blocks (15 problems in each block) with no time limit. During the last phase, each participant had to rate the cognitive load of MRRT and fluid intelligence test. The overall duration of Experiment 2 was about 80 min.

#### Design

Experiment 2 adopted a one-way between-subject experimental design (priming task type: generation and evaluation) with the priming task type as an independent variable. The dependent variable was the number of correct answers on the MRRT, and the mediating variables were relational mindset scores and cognitive load scores. The covariates were the participants’ ratings of the difficulty of the priming task and their fluid intelligence scores.

### Results

The independent sample *t*-tests showed that the total score on the MRRT was significantly higher in the generation group (*M* = 56.78, SD = 2.46) than in the evaluation group (*M* = 54.27, SD = 5.14), *t*_(129)_ = −3.60, *p* < 0.001, Cohen’s *d* = 0.63, indicating that generating distant analogies could effectively facilitate relational integration. Participants in the generation group (*M* = 11.58, SD = 3.62) reported more responses based on relational similarity than participants in the evaluation group (*M* = 8.91, SD = 4.72), *t*_(129)_ = −3.65, *p* < 0.001, Cohen’s *d* = 0.64, which suggested that generating distant analogies could elicit the relational mindset, making participants respond more based on relations. The main effects of priming tasks on extraneous cognitive load (*t*_(129)_ = 3.49, *p* < 0.001, Cohen’s *d* = 0.61), germane cognitive load (*t*_(129)_ = 2.07, *p* = 0.041, Cohen’s *d* = 0.36), and total cognitive load (*t*_(129)_ = 3.69, *p* < 0.001, Cohen’s *d* = 0.64) were significant. The total cognitive loads in the generation group (*M* = 30.81, SD = 6.11) were significantly lower than those in the evaluation group (*M* = 34.75, SD = 6.14), as we expected.

Before testing the mediation model, a correlation analysis was conducted among relational mindset, total cognitive loads, and scores on the MRRT. All of the bivariate correlations were statistically significant (Table 1). We took the priming task difficulty rating as a covariate to rule out the possible influence of mental effort differences among participants in the priming task. Participants’ fluid intelligence scores were utilized as an additional covariate to exclude the possible influence of participants’ differences in fluid intelligence on MRRT scores. Model 6 in PROCESS 3.0 (Hayes, 2017) was used to test the chain mediation model. The indirect effects were tested with bias-corrected bootstrapping (*n* = 5,000) and 95% confidence intervals (CI) for the indices.

**TABLE 1:** Correlation matrix in Experiment 2.

Variables names	1	2	3
(1) Relational mindset	–		
(2) Cognitive loads	−0.35***	–	
(3) MRRT scores	0.29***	−0.39***	–

***p < 0.001.

**FIGURE 2:**
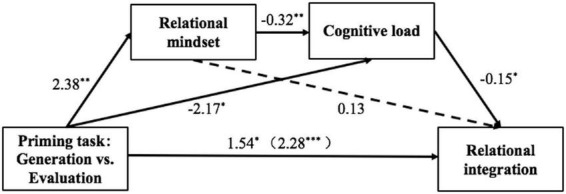
Mediation analysis of priming task and relational integration. *p < 0.05, **p < 0.01, ***p < 0.001.

Figure 2 depicts the results of the mediation analysis. The results of mediating effect analysis showed that the total effect of the priming task on relational integration was 2.28, SE = 0.69, *t* = 3.31, *p* = 0.001, 95% CI = [0.92, 3.64] (refer Table 2 for the regression model results and Table 3 for the effect values for each path). The direct effect was 1.54, SE = 0.70, *t* = 2.18, *p* = 0.031, 95% CI = [0.14, 2.93]. The total indirect effect was 0.74, SE = 0.37, 95% CI = [0.15, 1.61]. The indirect effects specifically included three paths, with the 95% confidence intervals of the indirect effects of two paths (priming task → cognitive load → relational integration and priming task → relational mindset → cognitive load → relational integration) not containing zero, indicating that these two indirect effects were significant, while the indirect effect of the priming task → relational mindset → relational integration path did not reach a significant level. In accordance with our prediction, generating distant analogies reduced cognitive load by inducing the relational mindset, thereby facilitating participants’ performance on relational integration.

**TABLE 2:** Regression estimates from the mediation model in Experiment 2.

Outcome	Predictors	*B*	SE	*t*	β
Relational integration	Difficulty rating	−0.32	0.22	−1.45	−0.12
	Fluid intelligence	0.67	0.18	3.64***	0.30
	Priming task	2.28	0.69	3.31***	0.55
	*R* ^2^	0.18			
	*F*	*F*_(3, 127)_ = 9.28, *p* < 0.001			
Relational mindset	Difficulty rating	−0.41	0.24	−1.71	−0.15
	Fluid intelligence	0.20	0.20	0.99	0.08
	Priming task	2.38	0.75	3.18**	0.54
	*R* ^2^	0.18			
	*F*	*F*_(3, 127)_ = 5.60, *p* = 0.001			
Cognitive load	Difficulty rating	1.27	0.32	4.02***	0.32
	Fluid intelligence	−1.02	0.26	−3.91***	−0.30
	Priming task	−2.17	1.01	−2.15*	−0.34
	Relational mindset	−0.32	0.12	−2.76**	−0.22
	*R* ^2^	0.31			
	*F*	*F*_(4, 126)_ = 13.98, *p* < 0.001			
Relational integration	Difficulty rating	−0.06	0.23	−0.26	−0.02
	Fluid intelligence	0.48	0.19	2.56*	0.22
	Priming task	1.54	0.70	2.18*	0.37
	Relational mindset	0.13	0.08	1.60	0.14
	Cognitive load	−0.15	0.06	−2.42*	−0.23
	*R* ^2^	0.25			
	*F*	*F*_(5, 125)_ = 8.14, *p* < 0.001			

Each column illustrates a regression model that predicts the criterion at the top of the column.

*p < 0.05, **p < 0.01, ***p < 0.001.

**TABLE 3:** Mediation effect estimates in Experiment 2.

	Indirect effect	Boot SE	Boot LLCI	Boot ULCI
Total indirect effect	0.74	0.37	0.15	1.61
Specific indirect effect				
Priming task → relational mindset → relational integration	0.31	0.34	−0.24	1.09
Priming task → cognitive load → relational integration	0.32	0.20	0.02	0.81
Priming task → relational mindset → cognitive load → relational integration	0.11	0.08	0.01	0.31

Values in bootstrapped confidence intervals (CIs) were based on 5,000 bootstrap draws.

SE = standard error.

### Discussion

The purpose of Experiment 2 was to explore the internal mechanism of the facilitation effect on relational integration by the distant analogy generation task using the MRRT with higher ecological validity. In comparison to the evaluation task, the generation task could elicit the relational mindset, lower cognitive load, and enhance participants’ relational reasoning performance. Importantly, relational mindset and cognitive load play a chain intermediary role in the internal mechanism of the facilitation effect, providing supporting evidence that generating distant analogies can serve as a priming task to promote relational integration and informing future research into the internal pathways of the facilitation effect.

## Experiment 3

Experiment 1 and Experiment 2 verified the facilitation effect of generating distant analogies on relational integration. In the practice, we hoped that the distant analogy generation task could be utilized as a warm-up exercise before learning, which could play a practical role in the subsequent learning tasks involving relational integration. Therefore, we investigated whether this facilitation effect also elicited in the real learning task in Experiment 3. We chose the interaction task in statistics mainly based on the following considerations. On the one hand, explaining interactions requires integrating information about all of the variables into a single complex concept, making this task suitable for examining relational integration. On the other hand, relational complexity theory holds that conceptual chunking into fewer, larger entities or segmentation into subtasks that can be operated on consecutively can reduce the amount of information that learners process in a single cognitive step (Halford et al., 1998). Cognitive strategies for conceptual chunking and segmentation are limited in interaction tasks due to the need to process multiple variables together. Therefore, we could examine the role of the distant analogy generation task in relational integration more purely on the premise that participants were less likely to employ cognitive strategies to reduce cognitive loads. We expected that learners in the distant analogy generation group would perform better on the interaction task.

### Materials and methods

#### Participants

A total of 86 college students were recruited, with 30 male students and 56 female students. Their mean age was 20.51 years (SD = 2.18, range: 18–27). We randomly allocated participants to the distant analogy generation group (GD; *n* = 42) and distant analogy evaluation group (ED; *n* = 44).

#### Materials

##### Priming task and fluid intelligence

The materials used in priming tasks (GD group and ED group) and the fluid intelligence task employed in Experiment 3 were the same as in Experiment 1a.

##### Interaction task

The interaction task was based on Halford et al. (2005). Multivariate interactions (i.e., interactions involving more than two independent variables) would result in high cognitive loads because the accurate interpretation of an interaction could not be based on a subset of variables, but rather required the concurrent consideration of multiple variables. The interaction task included two comparisons at different relational complexity levels: one was the comparison between binary interaction and ternary interaction, and the other was the comparison between ternary interaction and quaternary interaction. Participants needed to choose answers according to the interaction graphs with the topic of buying cakes. The given representations would have the lowest level of difference in pairs as a single entity, such as preference or difference (e.g., “*People prefer fresh cakes to frozen cakes*”). These sentences then described how other variables in the interaction affect this preference entity (e.g., “*The difference between fresh and frozen increases from chocolate cakes to carrot cakes*”). To keep the complexity of the task consistent across all interaction levels, we only used binary variables. Task materials were designed to be consistent across all of the other task characteristics except for relational complexity. To maintain a similar working memory load throughout the comparison, we balanced the amount of information expressed in words and the number of bars. Thus, two binary interactions would be compared to one ternary interaction, while two ternary interactions would be compared to one quaternary interaction.

##### Cognitive load questionnaire

The cognitive load scale employed in Experiment 3 was the same as that in Experiment 2. The three components showed good consistency reliability (Cronbach’s alpha = 0.69 for intrinsic cognitive load; Cronbach’s alpha = 0.73 for extraneous cognitive load; Cronbach’s alpha = 0.78 for germane cognitive load). Sum scores of each of the three cognitive load measures were obtained.

##### Prior knowledge

The question from Klepsch and Seufert (2021) and Xu et al. (2021) was used to assess participants’ prior knowledge on the interaction task (“How much did you know about the concept of “interaction” before the experiment started?” 1 = *very little*; 9 = *very much*).

##### Concept mastery scores

In accordance with Klepsch and Seufert (2021), we devised five questions to assess students’ comprehension of the interaction concept: (1) For the interaction task you just completed, to what extent do you feel you understand the task rules (1 = *not at all*; 9 = *very much*)? (2) Please briefly describe how the interaction task was accomplished. (3) Freshness (fresh/frozen), flavor (chocolate/carrot), type (iced/plain), and richness (rich/low fat) will affect people’s preferences for the cake. What are the independent and dependent variables? (4) A company produces a new beverage with five colors. If you want to examine the effect of colors on beverage sales, how many levels are there (A. 2, B. 3, C. 4, D. 5)? (5) Please describe briefly what “interaction” is and provide an example (not in this experiment). Questions 2 through 5 were worth five points each, out of 20 points. Two graduate students majoring in psychology were invited to rate students’ responses together with the experimenter. The ICC correlation coefficient value for the three raters was 0.79 (95% CI: 0.71–0.85, *p* < 0.001), indicating a high degree of measurement agreement for ratings. The concept mastery score for each student was calculated by taking the average of the three raters’ scores.

#### Procedure

Experiment 3 began with the same priming task as Experiment 2. The second task was the interaction task, presented with E-Prime 2.0.^1^ After the experimenter briefly introduced the background knowledge of the interaction concept, students were required to select the correct option according to the given premises and graphs. Options “A,” “B,” “C,” and “D” corresponded to “1,” “2,” “3,” and “4” on the keyboard, respectively. Problems included (1) a 2 × binary interaction example; (2) a 2 × binary interaction practice problem; (3) a ternary interaction example; (4) a ternary interaction practice problem; (5) four formal experimental problems, including two 2 × binary interaction problems and two ternary interaction problems, presented in random order; (6) a 2 × ternary interaction example; (7) a 2 × ternary interaction practice problem; (8) a quaternary interaction example; (9) a quaternary interaction practice problem; and (10) four formal experimental problems, including two 2 × ternary interaction problems and two quaternary interaction problems, presented in random order. The example presentation and practice stage were designed to provide sufficient training for the participants and familiarize them with the task rules. After each example and exercise was completed, textual representations, charts, and correct answers would reappear on the screen to provide feedback. No feedback was given in the formal experiment. Textual representations and graphs stayed on the screen until participants made their selection; thus, they were not required to keep additional information in working memory while solving the problem. The time spent solving each problem was recorded. Participants’ cognitive loads, prior knowledge, and concept mastery scores of the interaction were assessed following the interaction task. Finally, participants completed the fluid intelligence test. The overall duration of Experiment 3 was about 50 min.

#### Design

A two-way 2 (priming task type: generation, evaluation) × 2 (relational complexity: binary-ternary/ternary-quaternary) mixed experimental design was adopted, where the priming task type was the between-subject variable and the relational complexity was the within-subject variable. Cognitive loads, number of correct answers on the interaction task, reaction times, and concept mastery scores were dependent variables. Scores on fluid intelligence served as a covariate.

### Results

#### Prior knowledge

The independent sample *t*-test showed that there was no significant difference in prior knowledge between the generation group (*M* = 2.71, SD = 1.77) and the evaluation group (*M* = 3.25, SD = 2.14), *t*_(84)_ = 1.26, *p* = 0.210.

#### Cognitive loads

The main effect of the priming task type was significant for intrinsic cognitive load (*M*_GD_ = 10.72, SD = 2.00; *M*_ED_ = 11.58, SD = 1.88; *t*_(84)_ = 2.06, *p* = 0.042, Cohen’s *d* = 0.45) and extraneous cognitive load (*M*_GD_ = 11.52, SD = 3.09; *M*_ED_ = 12.87, SD = 2.90; *t*_(84)_ = 2.09, *p* = 0.040, Cohen’s *d* = 0.45), while the main effect on germane cognitive load was not significant (*M*_GD_ = 8.57, SD = 2.53; *M*_ED_ = 8.68, SD = 2.71; *t*_(84)_ = 0.20, *p* = 0.846). The total cognitive loads of the generation group (*M* = 30.81, SD = 5.70) were marginally lower than those of the evaluation group (*M* = 33.19, SD = 6.08), *t*_(84)_ = 1.87, *p* = 0.066, Cohen’s *d* = 0.40.

#### Number of correct answers on the interaction task

When assessing the effect of relational complexity and priming task type on the number of correct responses, we chose the generalized estimating equations model given that the dependent variable was an ordinal variable. Table 4 shows the number of participants responding in each condition. As the relational complexity increased, the number of participants responding correctly decreased, and the number of participants responding incorrectly increased.

**TABLE 4:** Number of participants responding in each condition in the interaction task.

		Relational complexity			
		2 × binary	Ternary	2 × ternary	Quaternary
Both correct	GD	36	31	35	24
	ED	39	34	28	26
One correct	GD	5	8	5	13
	ED	3	6	13	10
Both incorrect	GD	1	3	2	5
	ED	2	4	3	8

For the 2 × binary interaction task and the ternary interaction task, the coefficient of relational complexity (*B*) was 0.79, and *Exp (B)* was 2.21, *p* = 0.039. Participants were 2.21 times more likely to correctly answer the 2 × binary interaction questions than they were to correctly answer the ternary interaction questions. The coefficient for the priming task type (*B*) was 0.18 and *Exp (B)* was 1.19, *p* = 0.662. For the 2 × ternary interaction task and the quaternary interaction task, the coefficient of relational complexity (*B*) was 0.76, and *Exp (B)* was 2.13, *p* = 0.002, indicating that participants were 2.13 times more likely to answer the 2 × ternary interaction questions correctly than to answer the quaternary interaction questions correctly. The coefficient for the priming task type (*B*) was −0.46, and *Exp (B)* was 0.63, *p* = 0.216.

#### Solution times on the interaction task

**FIGURE 3:**
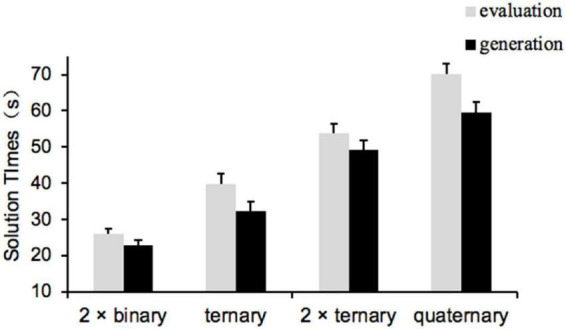
Mean solution times in Experiment 3. Error bars indicate standard errors.

Solution times were calculated across all trials. The repeated measures ANOVA revealed a significant main effect of relational complexity for the two binary interaction tasks and the ternary interaction task, *F*_(1_, _83)_ = 4.56, *p* = 0.036, partial η^2^ = 0.05. The solution times were significantly shorter for the 2 × binary interaction (*M* = 24.49, SE = 1.02) than for the ternary interaction (*M* = 36.04, SE = 1.97). The main effect of priming task type was significant, *F*_(1_, _83)_ = 4.05, *p* = 0.047, partial η^2^ = 0.05, with students in the generation group (*M* = 27.60, SE = 1.89) having significantly shorter solution times than those in the evaluation group (*M* = 32.94, SE = 1.85). The interaction between relational complexity and priming task type was not significant (*p* = 0.182). For the 2 × ternary interaction task and the quaternary interaction task, repeated measures ANOVA showed that the main effect of relational complexity was significant, *F*_(1_, _83)_ = 13.46, *p* < 0.001, partial η^2^ = 0.14. The solution times of 2 × ternary interaction (*M* = 51.56, SE = 1.76) were obviously shorter than those of quaternary interaction (*M* = 64.90, SE = 2.02). The main effect of priming task type was significant, *F*_(1_, _83)_ = 4.41, *p* = 0.039, partial η^2^ = 0.05. The solution times of the generation group (*M* = 54.47, SE = 2.56) were significantly shorter than those of the evaluation group (*M* = 62.00, SE = 2.50). Figure 3 shows a significant interaction between relational complexity and priming task type, *F*_(1_, _83)_ = 5.61, *p* = 0.020, partial η^2^ = 0.06. When participants performed the 2 × ternary interaction (lower relational complexity) problems, the difference in solution times between different priming tasks was not significant, *t*_(84)_ = 1.18, *p* = 1.000. The solution times of the generation group (*M* = 59.63, SE = 2.89) were substantially shorter than those of the evaluation group (*M* = 70.18, SE = 2.82) when participants completed quaternary interaction (higher relational complexity) problems, *t*_(84)_ = 2.77, *p* = 0.040, Cohen’s *d* = 0.30.

#### Concept mastery scores on the interaction task

The two priming task groups did not differ significantly in their understanding of task rules (*M*_GD_ = 7.40, SD = 1.50; *M*_ED_ = 7.52, SD = 1.46; *t*_(84)_ = 0.37, *p* = 0.712). Both groups of participants knew the task rules well, indicating that the results of the interaction task were reliable. The univariate ANOVA result showed that the main effect of the priming task type was significant (*F*_(1_, _83)_ = 4.34, *p* = 0.040, partial η^2^ = 0.05), with the concept mastery scores of the generation group (*M* = 14.99, SE = 0.38) being significantly higher than those of the evaluation group (*M* = 13.88, SE = 0.37).

### Discussion

In terms of prior knowledge of the interaction task, the two groups did not differ substantially and scored low (between 2 and 3 points), showing that they did not know much about the task before the experiment began and that any influence from prior knowledge could be ruled out. Regarding the understanding of the interaction task rules, the difference in scores between the two groups was not significant (both greater than seven points), indicating that students had a good grasp of task rules and could complete the task according to the rules. Consistent with Experiment 2, the generation task was successful in lowering students’ cognitive loads during learning since the cognitive load of the generation group was much lower than that of the evaluation group.

For the learning task we adopted, the interaction task, whether it was a comparison between 2 × binary interaction tasks and ternary interaction tasks or a comparison between 2 × ternary interaction tasks and quaternary interaction tasks, the relational complexity had a great impact on the number correct of tasks, which was consistent with Halford et al. (2005). However, the effect of priming task type on the number correct was not observed. In terms of solution times, in line with previous research (Halford et al., 2005), the more variables involved in the interaction task, the higher the relational complexity, and the longer the participants’ solution times. At the same time, the solution times of the participants in the generation group were significantly lower than those of the participants in the evaluation group, especially in the condition with the highest relational complexity (quaternary interaction), which had the greatest facilitation effect on the solution times. In addition to correct counts and solution times, we were interested in gaining insight into students’ mastery of the interaction concept. We discovered that compared with the distant evaluation group, students in the distant generation group had a deeper and more correct understanding of concepts. Completing the distant generation task in advance could not only improve the speed with which learners completed the task but also increase the depth of their understanding.

## General discussion

The purpose of this study was threefold: first, to verify the robustness of generating distant analogies to promote relational integration and to evaluate the possibility of evaluating distant analogies promoting relational integration; second, to explore the internal mechanism of the facilitation effect; and third, to examine the application of this effect in practical learning. We employed the classical n-term premise integration task (Experiment 1a) and the LST (Experiment 1b) to systematically compare the effects of generation and evaluation tasks on relational integration. Only the distant analogy generating task, and not the distant analogy evaluation task, enhanced the score in the n-term premise integration task (Experiment 1a) and the accuracy in the LST (Experiment 1b). In view of the domain generality of relational processing, the first two experiments offer integrative evidence for the facilitation effect of generating distant analogies. To improve the ecological validity of the study, we adopted an emergent MRRT in Experiment 2 to further corroborate the facilitation effect of the generation task and initially explore its internal mechanism. Consistent with the findings of Experiment 1a and 1b, Experiment 2 confirmed that the generation task facilitated relational integration better and further revealed that relational mindset and cognitive load played an intermediary role in the facilitation effect. In Experiment 3, we validated for the first time the facilitation effect in practical learning using a psychological statistical task, and we found that the distant generation task enhanced learners’ subsequent relational reasoning efficiency and improved their concept acquisition. Taken together, these results provide strong supporting evidence for the short-term facilitation of relational reasoning from the cognitive state by generating distant analogies, implying that the generation task can be used as an effective warm-up activity to facilitate learners’ performance on subsequent relational integration tasks involving unrelated material.

### Priming task and relational integration

Both Experiment 1a and 1b found that generating distant analogies facilitated relational integration compared to generating near analogies, which was in line with the findings of previous studies (Andrews and Bohadana, 2018; Andrews and Vann, 2019). This might be because near analogies can be solved by matching the same relation (e.g., a furnace burns coal, just like a woodstove burns wood), whereas completing distant analogies requires participants to generate a more abstract relation between two domains that are semantically distant [e.g., a furnace burns coal, just like the stomach “burns” (digests) food], which provides the experience of integrating relations across semantic distance, allowing individuals to better integrate relations in subsequent tasks. In exploring the facilitation effect of priming tasks, it is critical to set up an adequate control group for both the analogy generation task and the analogy evaluation task. We adopted a word generation task for our control group due to the absence of control groups in prior research (Andrews and Vann, 2019) or the large difference in the cognitive components included in the control and experimental groups (Andrews and Bohadana, 2018). We found no difference in performance between the control group (GC) and the distant analogy generation group (GD). This might be because the word generation task also required participants to explore the relations between words, involving a certain process of relational integration, which could be further explored in the future to determine what types of activities are better suited as control tasks.

Consistent with the only study that compared the facilitation effects of distant analogy generation and distant analogy evaluation (Vendetti et al., 2014), we found that the generation task improved participants’ performance on relational integration compared to the evaluation task. Despite the fact that both priming tasks entailed distant relational processing, the facilitation effect was stronger for the distant analogy generation task. This may be because participants needed to retrieve alternative options based on the extracted semantic relations and the item C and to assess the validity of the options in solving the generative problem. In contrast, evaluating a complete analogy problem required merely a comparison of relations and did not involve an active search for solutions to complete the analogy as a processing step. Thus, the distant analogy generation task was comparatively more effective in eliciting the experience that could be transferred to subsequent tasks, showing the generation task’s uniqueness and significance.

### Internal mechanism of the facilitation effect

Consistent with previous research, we observed that participants in the generation group reported a stronger relational mindset. The relational mindset emerged when individuals actively identified and constructed similar relations, leading to more relational responses in subsequent tasks (Brown and Kane, 1988; Goldwater and Markman, 2011). The results were in line with those of Vendetti et al. (2014). Although both priming tasks involved analogical processing at distant semantic distances, the generation task elicited a stronger relational mindset. This may be because the generation task required participants to generate answers actively. In contrast, the problems of the evaluation task offered participants solutions and accordingly did not strongly activate individual knowledge about relations.

The present study is the first to indicate that generating distant analogies can lessen individuals’ total cognitive loads. Although participants in both experimental groups completed the same relational integration task, participants in the generation group experienced less cognitive load, which was consistent with our expectations. We also found that the generation group reduced participants’ germane cognitive load and extraneous cognitive load.

According to cognitive load theory, higher germane cognitive loads indicate that participants put in more mental effort and perform better in tasks (Kühl and Eitel, 2016). However, our results showed that the evaluation group with higher germane cognitive load did not perform well as the generation group. Possible reasons for this are that participants were influenced by the uncertainty of the priming task and their attribution style. First, some participants reported that the answers to the evaluation task were not unique and would change depending on their understanding and interpretation, making them feel uncertain and ambiguous. This uncertainty might increase participants’ cognitive loads and compete for cognitive resources with later relational integration tasks, resulting in a negative impact on task performance, compared to the analogy generation task with definitive solutions (Moran, 2016). Second, if individuals felt that the task difficulty factor was beyond their control, they would not exert extra effort accordingly (Weiner, 2010), resulting in their poor performance on relational integration.

Solving the distant generation task required the exclusion of irrelevant answers when generating answers, which could improve participants’ capacity to suppress irrelevant information and reduce extraneous cognitive load. When generating analogies, participants needed to identify and select appropriate relations and item D through controlled semantic retrieval and inhibitory control. However, when evaluating analogies, participants simply needed to judge whether the given relations were valid in the confined search space. There was no need to exclude distractions, so the requirements for controlling semantic extraction and inhibitory control were not very high. Andrews and Vann (2019) found that participants in the distant analogy generation group could better inhibit belief-based responding and facilitate relational processing of valid but unbelievable problems, possibly because generating distant analogies facilitated inhibitory control and enabled participants to integrate relations between premises better. Future research can look into whether the distant analogy generation task could promote inhibitory control.

Interventions that encourage learners to pay attention to relations tend to promote learning (Goldwater and Schalk, 2016). We found that the generation task could facilitate participants’ relational integration performance by inducing the relational mindset and reducing total cognitive loads. Compared to passively evaluating analogies, actively generating answers to analogies may activate individuals’ prior knowledge about relations through pre-training (Kapur and Bielaczyc, 2012), making the integration of new information and available knowledge easier (Toh and Kapur, 2017), reducing the working memory load originally needed, and thus making relational reasoning more efficient. It is worth mentioning that simply eliciting a relational mindset was not enough for the priming task to enhance relational integration. The priming task would fail to achieve the facilitation if it merely introduced a relational mindset without further reducing cognitive loads. Thus, the present study tentatively found that cognitive load played a decisive role in the underlying mechanism of the facilitation effect.

### Preliminary application of the facilitation effect in learning

The key to teaching higher-order thinking in disciplines such as mathematics, science, and history is to conceptualize learning as a system of developing and operationalizing relationships. Educators need to provide appropriate support to draw students’ attention to relational representations of problems and concepts (Richland and Simms, 2015). Given that we discovered that generating distant analogies produced a robust facilitation effect on relational integration in Experiment 1 and 2, we then verified the facilitation effect in the learning setting in Experiment 3 for the first time. Considering that the students may have backgrounds in the humanities or natural sciences and that their prior knowledge may differ significantly, we selected an important statistics concept, the interaction effect, to ensure that the conclusions were highly relevant to school education. We discovered that students who were in the generation group took less time and had a deeper understanding of the interaction without compromising correctness than those who were in the evaluation group.

The facilitation of relational reasoning observed in the present research has potential implications for non-laboratory settings. In the future, having students tackle distant analogy problems before introducing complicated concepts to them may assist them in gaining an initial understanding, which can then be consolidated and elaborated through example exercises. The temporary tendency toward relational thinking induced by distant analogies may develop into a more stable and enduring state of thinking relationally after warming up in this and other situations.

### Limitations and future research

Although we provide evidence for the facilitation effect on relational integration of generating distant analogies and initially explore the internal mechanism of the facilitation effect through three laboratory experiments, there is still potential for improvement, which can be refined in future research. First, it will be imperative to systematically develop and test the effectiveness of interventions for relational reasoning outside the laboratory in the future, allowing for a deeper understanding of what students have learned by critically comparing, pointing out differences, presenting counterarguments, or perceiving contradictions (Alexander, 2019).

Second, the present study only provides a preliminary exploration of the facilitation effect’s internal mechanism. The impact of other factors, such as inhibitory control and motivational factors, on the facilitation effect can be further explored in the future. Subjective and objective measures of motivation levels during the task can be incorporated in the future to help educators confirm the role played by motivational factors, because learners may be more motivated and engaged during the exploration and generation process involved in the distant analogy generation task (Kapur, 2016).

Third, there are possible limitations of using subjective ratings to measure cognitive load; for example, the subjective rating scores may be influenced by the participant’s understanding of the item (Zheng, 2017). In addition to traditional subjective measures (offline), there are also emerging objective measures (online) for measuring cognitive load when participants complete the task, such as recording eye movement data (Chen and Epps, 2013), heart rate variability (Durantin et al., 2014) or functional near-infrared spectroscopy (Fishburn et al., 2014). In the future, online measurement of physiological data can be used to measure participants’ instantaneous cognitive load and provide more valid evidence for the mediation effect.

## Conclusion

The present experiments establish the robustness of the facilitation effect for generating distant analogies, but not evaluating distant analogies, providing convergent evidence by adopting three various relational integration paradigms. Our research also reveals that completing the distant analogy generation task would induce learners’ relational mindset, reduce cognitive loads, and facilitate relational integration performance compared to completing the distant analogy evaluation task. To our knowledge, the present research is the first to examine the preliminary role of the facilitation effect in practical learning. Educators can attempt to employ distant analogy generation as an effective warm-up activity in future instructional interventions to initiate students’ relational integration from the state.

## Data availability statement

The raw data supporting the conclusions of this article will be made available by the authors, without undue reservation.

## Ethics statement

The studies involving human participants were reviewed and approved by Institutional Review Board of Tsinghua University, Department of Psychology. The patients/participants provided their written informed consent to participate in this study.

## Author contributions

XD: literature review, design and execution of experiments, formal analysis, discussion, and writing. PS: resources, supervision, and editing. Both authors have read and agreed to the final version of the manuscript for submission.

## Footnotes

1
www.pstnet.com

## References

[B1] Alexander P. A. (2019). Individual differences in college-age learners: The importance of relational reasoning for learning and assessment in higher education. *Brit. J. Educ. Psychol.* 89 416–428. 10.1111/bjep.12264 30632610

[B2] Alexander P. A. Dumas D. Grossnickle E. M. List A. Firetto C. M. (2016a). Measuring relational reasoning. *J. Exp. Educ.* 84 119–151. 10.1080/00220973.2014.963216

[B3] Alexander P. A. Singer L. M. Jablansky S. Hattan C. (2016b). Relational reasoning in word and in figure. *J. Educ. Psychol.* 108 1140–1152. 10.1037/edu0000110

[B4] Andrews G. Birney D. Halford G. S. (2006). Relational processing and working memory capacity in comprehension of relative clause sentences. *Mem. Cogn.* 34 1325–1340. 10.3758/bf03193275 17225512

[B5] Andrews G. Bohadana G. R. (2018). Does solving distant analogies facilitate relational integration? *J. Cogn. Psychol.* 30 270–280. 10.1080/20445911.2017.1414223

[B6] Andrews G. Halford G. S. Shum D. Maujean A. Chappell M. Birney D. (2013). Relational processing following stroke. *Brain Cogn.* 81 44–51. 10.1016/j.bandc.2012.09.003 23174427

[B7] Andrews G. Mihelic M. (2014). Belief-based and analytic processing in transitive inference: Further evidence for the importance of premise integration. *J. Cogn. Psychol.* 26 588–596. 10.1080/20445911.2014.909434

[B8] Andrews G. Vann D. M. (2019). Solving distant analogies reduces belief-based responding in transitive inference. *J. Cogn. Psychol.* 31 760–767. 10.1080/20445911.2019.1657432

[B9] Arthur W. Jr. Tubre T. C. Paul D. S. Sanchez-Ku M. L. (1999). College-sample psychometric and normative data on a short form of the Raven Advanced Progressive Matrices Test. *J. Psychoeduc. Assess.* 17 354–361. 10.1177/073428299901700405

[B10] Birney D. P. Halford G. S. Andrews G. (2006). Measuring the influence of complexity on relational reasoning: The development of the Latin Square Task. *Educ. Psychol. Meas.* 66 146–171. 10.1177/0013164405278570

[B11] Bliznashki S. Kokinov B. (2010). “Relational versus attributional mode of problem solving?,” in *Proceedings of the Thirty-Second Annual Conference of the Cognitive Science Society (Portland).*

[B12] Braasch J. L. Goldman S. R. Wiley J. (2013). The influences of text and reader characteristics on learning from refutations in science texts. *J. Educ. Psychol.* 105 561–578. 10.1037/a0032627

[B13] Brainard D. H. Vision S. (1997). The psychophysics toolbox. *Spatial Vis.* 10 433–436. 10.1163/156856897x00357 9176952

[B14] Brown A. L. Kane M. J. (1988). Preschool children can learn to transfer: Learning to learn and learning from example. *Cogn. Psychol.* 20 493–523. 10.1016/0010-0285(88)90014-x 3191668

[B15] Bunch K. M. Andrews G. Halford G. S. (2007). Complexity effects on the children’s gambling task. *Cogn. Dev.* 22 376–383. 10.1016/j.cogdev.2007.01.004

[B16] Chen S. Epps J. (2013). Automatic classification of eye activity for cognitive load measurement with emotion interference. *Comput. Meth. Prog. Bio.* 110 111–124. 10.1016/j.cmpb.2012.10.021 23270963

[B17] Cortes R. A. Weinberger A. B. Colaizzi G. A. Porter G. Dyke E. Keaton H. (2021). What Makes Mental Modeling Difficult? Normative Data for the Multidimensional Relational Reasoning Task (MRRT). *Front. Psychol.* 12:1512. 10.3389/fpsyg.2021.668256 34025531 PMC8134533

[B18] Dumas D. Dong Y. (2019). Development and calibration of the student opportunities for deeper learning instrument. *Psychol. Schools* 56 1381–1412. 10.1002/pits.22292

[B19] Durantin G. Gagnon J. F. Tremblay S. Dehais F. (2014). Using near infrared spectroscopy and heart rate variability to detect mental overload. *Behav. Brain Res.* 259 16–23. 10.1016/j.bbr.2013.10.042 24184083

[B20] English L. D. Halford G. S. (2012). *Mathematics Education: Models and Processes.* New York, NY: Routledge, 10.4324/9780203052884

[B21] Fishburn F. A. Norr M. E. Medvedev A. V. Vaidya C. J. (2014). Sensitivity of fNIRS to cognitive state and load. *Front. Hum. Neurosci.* 8:76. 10.3389/fnhum.2014.00076 24600374 PMC3930096

[B22] Gentner D. (2016). Language as cognitive tool kit: How language supports relational thought. *Am. Psychol.* 71 650–657. 10.1037/amp0000082 27977235

[B23] Gick M. L. Holyoak K. J. (1980). Analogical problem solving. *Cogn. Psychol.* 12 306–355.

[B24] Goldstone R. L. Medin D. L. Gentner D. (1991). Relational similarity and the nonindependence of features in similarity judgments. *Cogn. Psychol.* 23 222–262. 10.1016/0010-0285(91)90010-l 2055001

[B25] Goldwater M. B. Jamrozik A. (2019). Can a relational mindset boost analogical retrieval? *Cogn. Res.* 4:47. 10.1186/s41235-019-0198-8 31858283 PMC6923295

[B26] Goldwater M. B. Markman A. B. (2011). Categorizing entities by common role. *Psychon. B. Rev.* 18 406–413. 10.3758/s13423-011-0058-0 21327374

[B27] Goldwater M. B. Schalk L. (2016). Relational categories as a bridge between cognitive and educational research. *Psychol. Bull.* 142 729–757. 10.1037/bul0000043 26950007

[B28] Gray M. E. Holyoak K. J. (2020). Individual differences in relational reasoning. *Mem. Cogn.* 48 96–110. 10.3758/s13421-019-00964-y 31317394

[B29] Gray M. E. Holyoak K. J. (2021). Teaching by analogy: From theory to practice. *Mind Brain Educ.* 15 250–263. 10.1111/mbe.12288

[B30] Green A. E. (2017). Creativity, within reason: Semantic distance and dynamic state creativity in relational thinking and reasoning. *Curr. Dir. Psychol. Sci.* 25 28–35. 10.1177/0963721415618485

[B31] Green A. E. Kraemer D. J. Fugelsang J. A. Gray J. R. Dunbar K. N. (2010). Connecting long distance: Semantic distance in analogical reasoning modulates frontopolar cortex activity. *Cereb. Cortex* 20 70–76. 10.1093/cercor/bhp081 19383937

[B32] Green A. E. Kraemer D. J. Fugelsang J. A. Gray J. R. Dunbar K. N. (2012). Neural correlates of creativity in analogical reasoning. *J. Exp. Psychol. Learn.* 38 264–272. 10.1037/a0025764 22103784

[B33] Green A. E. Spiegel K. A. Giangrande E. J. Weinberger A. B. Gallagher N. M. Turkeltaub P. E. (2017). Thinking cap plus thinking zap: tDCS of frontopolar cortex improves creative analogical reasoning and facilitates conscious augmentation of state creativity in verb generation. *Cereb. Cortex* 27 2628–2639. 10.1093/cercor/bhw080 27075035 PMC6361291

[B34] Halford G. S. Baker R. McCredden J. E. Bain J. D. (2005). How many variables can humans process? *Psychol. Sci.* 16 70–76. 10.1111/j.0956-7976.2005.00782.x 15660854

[B35] Halford G. S. Wilson W. H. Phillips S. (1998). Processing capacity defined by relational complexity: Implications for comparative, developmental, and cognitive psychology. *Behav. Brain Sci.* 21 803–831. 10.1017/s0140525x98001769 10191879

[B36] Hansell N. K. Halford G. S. Andrews G. Shum D. H. Harris S. E. Davies G. (2015). Genetic basis of a cognitive complexity metric. *PLoS. One.* 10:e0123886. 10.1371/journal.pone.0123886 25860228 PMC4393228

[B37] Hayes A. F. (2017). *Introduction to Mediation, Moderation, and Conditional Process Analysis: A Regression-Based Approach.* Now York, NY: Guilford publications.

[B38] Hearne L. J. Birney D. P. Cocchi L. Mattingley J. B. (2020). The Latin Square Task as a Measure of Relational Reasoning. *Eur. J. Psychol. Assess.* 36 296–302. 10.1027/1015-5759/a000520

[B39] Holyoak K. J. Thagard P. (1996). *Mental leaps: Analogy in Creative Thought.* Cambridge, MA: The Mit Press.

[B40] James W. (1890). *The Principles of Psychology.* New York, NY: Henry Holt and Company.

[B41] Kalkstein D. A. Hackel L. M. Trope Y. (2020). Person-centered cognition: The presence of people in a visual scene promotes relational reasoning. *J. Exp. Soc. Psychol.* 90:104009. 10.1016/j.jesp.2020.104009

[B42] Kalyuga S. (2011). Cognitive load theory: How many types of load does it really need? *Educ. Psychol. Rev.* 23 1–19. 10.1007/s10648-010-9150-7

[B43] Kapur M. (2016). Examining productive failure, productive success, unproductive failure, and unproductive success in learning. *Educ. Psychol.* 51 289–299. 10.1080/00461520.2016.1155457

[B44] Kapur M. Bielaczyc K. (2012). Designing for productive failure. *J. Learn. Sci.* 21, 45–83. 10.1080/10508406.2011.591717

[B45] Klepsch M. Schmitz F. Seufert T. (2017). Development and validation of two instruments measuring intrinsic, extraneous, and germane cognitive load. *Front. Psychol.* 8:1997. 10.3389/fpsyg.2017.01997 29201011 PMC5696680

[B46] Klepsch M. Seufert T. (2021). Making an effort versus experiencing load. *Front. Educ.* 6:645284. 10.3389/feduc.2021.645284

[B47] Knowlton B. J. Morrison R. G. Hummel J. E. Holyoak K. J. (2012). A neurocomputational system for relational reasoning. *Trends Cogn. Sci.* 16 373–381. 10.1016/j.tics.2012.06.002 22717468

[B48] Kühl T. Eitel A. (2016). Effects of disfluency on cognitive and metacognitive processes and outcomes. *Metacogn. Learn.* 11 1–13. 10.1007/s11409-016-9154-x

[B49] Markman A. B. Gentner D. (1993). Structural alignment during similarity comparisons. *Cogn. Psychol.* 25 431–467. 10.1006/cogp.1993.1011

[B50] Moran T. P. (2016). Anxiety and working memory capacity: A meta-analysis and narrative review. *Psychol. Bull.* 142 831–864. 10.1037/bul0000051 26963369

[B51] Murphy A. N. Zheng Y. Shivaram A. Vollman E. Richland L. E. (2021). Bias and sensitivity to task constraints in spontaneous relational attention. *J. Exp. Child Psychol.* 202:104981. 10.1016/j.jecp.2020.104981 33161340

[B52] Richland L. E. Simms N. (2015). Analogy, higher order thinking, and education. *Wires. Cogn. Sci.* 6 177–192. 10.1002/wcs.1336 26263071

[B53] Simms N. K. Frausel R. R. Richland L. E. (2018). Working memory predicts children’s analogical reasoning. *J. Exp. Child Psychol.* 166 160–177. 10.1016/j.jecp.2017.08.005 28923594

[B54] Simms N. K. Richland L. E. (2019). Generating relations elicits a relational mindset in children. *Cogn. Sci.* 43:e12795. 10.1111/cogs.12795 31621120

[B55] Singley A. T. M. Bunge S. A. (2014). Neurodevelopment of relational reasoning: Implications for mathematical pedagogy. *Trends Neurosci. Educ.* 3 33–37. 10.1016/j.tine.2014.03.001

[B56] Sternberg R. J. (1977). Component processes in analogical reasoning. *Psychol. Rev.* 84 353–378. 10.1037/0033-295x.84.4.353

[B57] Sweller J. (1994). Cognitive load theory, learning difficulty, and instructional design. *Learn. Instr.* 4 295–312. 10.1016/0959-4752(94)90003-5

[B58] Sweller J. (2010). Element interactivity and intrinsic, extraneous, and germane cognitive load. *Educ. Psychol. Rev.* 22 123–138. 10.1007/s10648-010-9128-5

[B59] Sweller J. Ayres P. Kalyuga S. (2011). *Cognitive Load Theory.* New York, NY: Springer.

[B60] Sweller J. Sweller S. (2006). Natural information processing systems. *Evol. Psychol.* 4:147470490600400135. 10.1177/147470490600400135

[B61] Sweller J. van Merriënboer J. Paas F. G. (1998). Cognitive architecture and instructional design. *Educ. Psychol. Rev.* 10 251–296.

[B62] Sweller J. van Merriënboer J. Paas F. G. (2019). Cognitive architecture and instructional design: 20 years later. *Educ. Psychol. Rev.* 31 261–292. 10.1007/s10648-019-09465-5

[B63] Toh P. L. Kapur M. (2017). Is having more prerequisite knowledge better for learning from productive failure? *Instr. Sci.* 45 377–394. 10.1007/s11251-016-9402-0

[B64] Tohill J. M. Holyoak K. J. (2000). The impact of anxiety on analogical reasoning. *Think. Reason.* 6 27–40. 10.1080/135467800393911

[B65] Vendetti M. S. Matlen B. J. Richland L. E. Bunge S. A. (2015). Analogical reasoning in the classroom: Insights from cognitive science. *Mind Brain Educ.* 9 100–106. 10.1111/mbe.12080

[B66] Vendetti M. S. Wu A. Holyoak K. J. (2014). Far-out thinking: Generating solutions to distant analogies promotes relational thinking. *Psychol. Sci.* 25 928–933. 10.1177/0956797613518079 24463552

[B67] Weinberger A. B. Iyer H. Green A. E. (2016). Conscious augmentation of creative state enhances “real” creativity in open-ended analogical reasoning. *PLoS. One* 11:e0150773. 10.1371/journal.pone.0150773 26959821 PMC4784911

[B68] Weiner B. (2010). The development of an attribution-based theory of motivation: A history of ideas. *Educ. Psychol.* 45 28–36. 10.1080/00461520903433596

[B69] Whitaker K. J. Vendetti M. S. Wendelken C. Bunge S. A. (2017). Neuroscientific insights into the development of analogical reasoning. *Dev. Sci.* 21:e12531. 10.1111/desc.12531 28295877 PMC5887920

[B70] Xu K. M. Koorn P. de Koning B. Skuballa I. T. Lin L. Henderikx M. (2021). A growth mindset lowers perceived cognitive load and improves learning: Integrating motivation to cognitive load. *J. Educ. Psychol.* 113 1177–1191. 10.1037/edu0000631

[B71] Zheng R. Z. (2017). *Cognitive Load Measurement and Application.* New York, NY: Routledge, 10.4324/9781315296258

